# Hospital Readmissions in Patients Supported with Durable Centrifugal-Flow Left Ventricular Assist Devices

**DOI:** 10.3390/jcm13102869

**Published:** 2024-05-13

**Authors:** Christos P. Kyriakopoulos, Craig H. Selzman, Theodoros V. Giannouchos, Rohan Mylavarapu, Konstantinos Sideris, Ashley Elmer, Nathan Vance, Thomas C. Hanff, Hiroshi Kagawa, Josef Stehlik, Stavros G. Drakos, Matthew L. Goodwin

**Affiliations:** 1 Division of Cardiovascular Medicine, Department of Internal Medicine, University of Utah Health and School of Medicine, Salt Lake City, UT 84132, USA; christos.kyriakopoulos@hsc.utah.edu (C.P.K.); konstantinos.sideris@hsc.utah.edu (K.S.); thomas.hanff@hsc.utah.edu (T.C.H.); josef.stehlik@hsc.utah.edu (J.S.); stavros.drakos@hsc.utah.edu (S.G.D.); 2 George E. Wahlen Department of Veterans Affairs Medical Center, Salt Lake City, UT 84148, USA; craig.selzman@hsc.utah.edu (C.H.S.); rohan.mylavarapu@hsc.utah.edu (R.M.); ashley.elmer@hsc.utah.edu (A.E.); nathan.vance@hsc.utah.edu (N.V.); hiroshi.kagawa@hsc.utah.edu (H.K.); 3 Nora Eccles Harrison Cardiovascular Research and Training Institute, University of Utah, Salt Lake City, UT 84112, USA; 4 Division of Cardiothoracic Surgery, Department of Surgery, University of Utah Health and School of Medicine, Salt Lake City, UT 84132, USA; 5 Department of Health Policy & Organization, School of Public Health, The University of Alabama at Birmingham, Birmingham, AL 35294, USA; tgiannou@uab.edu

**Keywords:** heart failure, left ventricular assist device, hospital readmission, hospitalization

## Abstract

**Background:** Centrifugal-flow left ventricular assist devices (CF-LVADs) have improved morbidity and mortality for their recipients. Hospital readmissions remain common, negatively impacting quality of life and survival. We sought to identify risk factors associated with hospital readmissions among patients with CF-LVADs. **Methods**: Consecutive patients receiving a CF-LVAD between February 2011 and March 2021 were retrospectively evaluated using prospectively maintained institutional databases. Hospital readmissions within three years post-LVAD implantation were dichotomized into heart failure (HF)/LVAD-related or non-HF/LVAD-related readmissions. Multivariable Cox regression models augmented using a machine learning algorithm, the least absolute shrinkage and selection operator (LASSO) method, for variable selection were used to estimate associations between HF/LVAD-related readmissions and pre-, intra- and post-operative clinical variables. **Results:** A total of 204 CF-LVAD recipients were included, of which 138 (67.7%) had at least one HF/LVAD-related readmission. HF/LVAD-related readmissions accounted for 74.4% (436/586) of total readmissions. The main reasons for HF/LVAD-related readmissions were major bleeding, major infection, HF exacerbation, and neurological dysfunction. Using pre-LVAD variables, HF/LVAD-related readmissions were associated with substance use, previous cardiac surgery, HF duration, pre-LVAD inotrope dependence, percutaneous LVAD/VA-ECMO support, LVAD type, and the left ventricular ejection fraction in multivariable analysis (Harrell’s concordance c-statistic; 0.629). After adding intra- and post-operative variables in the multivariable model, LVAD implant hospitalization length of stay was an additional predictor of readmission. **Conclusions:** Using machine learning-based techniques, we generated models identifying pre-, intra-, and post-operative variables associated with a higher likelihood of rehospitalizations among patients on CF-LVAD support. These models could provide guidance in identifying patients with increased readmission risk for whom clinical strategies to mitigate this risk may further improve LVAD recipient outcomes.

## 1. Introduction

Durable mechanical circulatory support (MCS) is an established and increasingly employed therapeutic approach for patients with advanced heart failure (HF) [1]. The advent of the newer generation of centrifugal-flow left ventricular assist devices (LVADs) has significantly improved adverse event rates and survival compared to older axial-flow devices [1]. Nevertheless, hospital readmissions following LVAD implantation remain a common and vexing clinical issue adversely affecting patient quality of life and survival, as well as increasing healthcare costs and resource utilization [1,2,3,4]. It has been reported that up to 71% of patients are rehospitalized within one year following LVAD implantation [1,3,4,5,6].

The implementation of a new heart allocation policy by the Organ Procurement and Transplantation Network in the United States prioritizing the use of temporary MCS devices as a bridge to transplantation (BTT) method, portending a higher priority status, has led to a significant decrease in the number of LVADs implanted as BTT [7]. With a gradual shift towards the use of LVADs as destination therapy (DT), it is crucial to identify clinical factors associated with rehospitalizations to potentially ameliorate their burden on patients, healthcare providers, and healthcare systems.

Previous efforts investigating rehospitalization while on LVAD support focused on the early period following device implantation [1,3,4,5,6]. While this period is crucial, it is also important to investigate the reasons for readmission at later timepoints, especially given the increasing use of durable LVADs as DT [1]. In this context, we sought to explore HF- or LVAD-related hospital readmissions and their association with sociodemographic, pre-, intra-, and post-operative clinical variables in a contemporary cohort of patients on centrifugal-flow LVAD support. The identification of risk factors associated with a higher risk of rehospitalization could guide the implementation of appropriate therapeutic strategies to mitigate this risk and improve LVAD recipient outcomes.

## 2. Methods

### 2.1. Study Population

Advanced HF patients receiving a centrifugal-flow LVAD between February 2011 and March 2021 at the University of Utah Hospital or the George E. Wahlen Department of Veterans Affairs Medical Center, both in Salt Lake City, Utah, were retrospectively evaluated using prospectively maintained institutional databases. Patients were followed until LVAD explantation due to heart transplantation or cardiac recovery, death, three years following LVAD implantation hospital discharge, or the study’s conclusion in March 2022. Patients who died prior to hospital discharge following LVAD implantation were excluded from the analysis. The study was approved by the University of Utah Institutional Review Board overseeing research studies taking place at both institutions and written informed consent was obtained from all patients (Approval Code: 30622; most recent approval date was 9 February 2024).

### 2.2. Clinical Management and Definitions

Data collection included demographics, comorbidities, psychosocial factors, HF etiology and duration, the use of guideline-directed HF medical therapy, laboratory data, hemodynamic data obtained via right heart catheterization (RHC) and echocardiographic data obtained prior to and closest to the LVAD implantation, as well as intraoperative data. Guideline-directed HF medical therapy and RHC-derived hemodynamic data were collected two to four months following LVAD implantation, with at least two consecutive weeks being required as a minimum for a patient to be considered as being on a specific pharmacologic agent therapy.

HF duration was defined as the time from HF symptom onset to LVAD implantation, as ascertained through chart review. The effect of LVAD unloading on cardiac size, shape, and function was assessed via echocardiography and invasive hemodynamic measurements following LVAD implantation and prior to discharge. LVAD speed was adjusted to optimize blood flow and left ventricular decompression with positioning of the interventricular and interatrial septa in the midline, minimal mitral valve regurgitation, and intermittent aortic valve opening, in order of decreasing priority. Subsequent speed adjustments were made as indicated by patient symptoms and/or clinical events. Patients were medically managed at the discretion of the treating physicians within the participating institutions as per the established standard HF therapy guidelines.

### 2.3. Hospital Readmission Categorization

Hospital readmissions within three years post-LVAD hospitalization discharge to the implanting hospitals or an outlying hospital were prospectively recorded and were retrospectively reviewed for adjudication of the readmission reason (C.P.K., M.L.G.). Readmissions were categorized into the following categories based on the Society of Thoracic Surgeons Interagency Registry for Mechanically Assisted Circulatory Support (INTERMACS) categorization [1]: major bleeding, major infection, HF exacerbation, cardiac arrhythmia, device malfunction, neurological dysfunction, gastrointestinal disorder, anticoagulant adjustment, respiratory distress/failure, trauma, electrolyte abnormalities, endocrine disorder, acute or acute on chronic renal failure, pre-syncope/syncope, hypovolemia/hypotension, chest pain, planned procedure, or other. Hospital readmissions were subsequently dichotomized into HF/LVAD-related or non-HF/LVAD-related readmissions. Readmissions related to gastrointestinal disorders, respiratory distress/failure, trauma, endocrine disorders, chest pain, planned procedure, or other, were classified as non-HF/LVAD-related readmissions (Table 1).

### 2.4. Study Outcomes

The primary outcome of interest was the incidence of any HF/LVAD-related readmission up to three years post-LVAD implantation hospital discharge. The secondary outcome was the hazard of HF/LVAD-related readmissions while on LVAD support over the three years post-LVAD implantation hospital discharge.

### 2.5. Statistical Analysis

Baseline clinical characteristics were summarized using standard summary statistics including frequencies, percentages, and means. Measures of variation were presented as the mean ± standard deviation. Differences between patient subgroups for categorical variables were evaluated using Fisher’s exact test and continuous variables were evaluated using the two-group Student’s *t*-test or Kruskal–Wallis test, depending on the normality of the distribution.

We estimated the associations between having at least one HF/LVAD-related readmission within three years post-LVAD implantation hospital discharge and major independent sociodemographic and clinical variables. We used multivariable Cox regression models augmented using a machine learning algorithm, the least absolute shrinkage and selection operator (LASSO) method, for variable selection and regularization, similar to previous work [8,9]. Two models were generated, with the one employing only baseline pre-LVAD implantation variables and the second also including intraoperative and post-LVAD implantation variables. The predictive accuracy of the models for HF/LVAD-related readmissions was evaluated using Harrell’s concordance c-statistic and the hazard curve of HF/LVAD-related readmissions up to three years post-LVAD implantation hospital discharge was generated. A *p*-value of <0.05 was considered statistically significant and all reported *p*-values were two-tailed. All analyses were performed using STATA 17.0 (StataCorp, College Station, TX, USA).

## 3. Results

**Figure 1:**
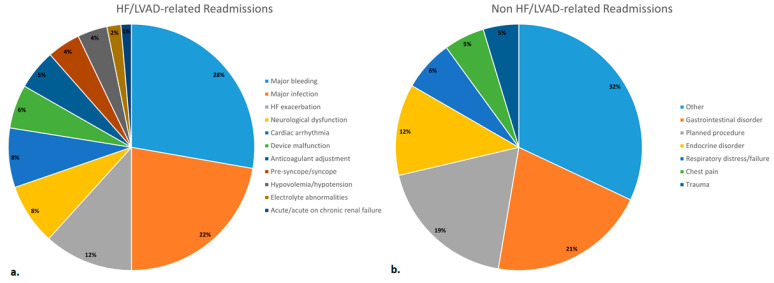
Rates of (**a**) heart failure/left ventricular assist device-related and (**b**) non-heart failure/left ventricular assist device-related readmissions.

Overall, 222 patients receiving a centrifugal-flow LVAD were prospectively enrolled over the study period. Fifteen patients who died prior to hospital discharge following LVAD implantation were excluded (6.8%). Additionally, three patients were excluded due to incomplete data (1.4%). This resulted in a final cohort of 204 patients, and 586 readmissions were observed during the study period. HF- or LVAD-related reasons accounted for 436 out of the 586 readmissions. HF/LVAD-related readmissions were mainly for major bleeding (27.8%), major infection (22.2%), HF exacerbation (11.7%), and neurological dysfunction (8.0%). The rates of HF/LVAD and non-HF/LVAD-related reasons for readmission are presented in Figure 1.

**Figure 2:**
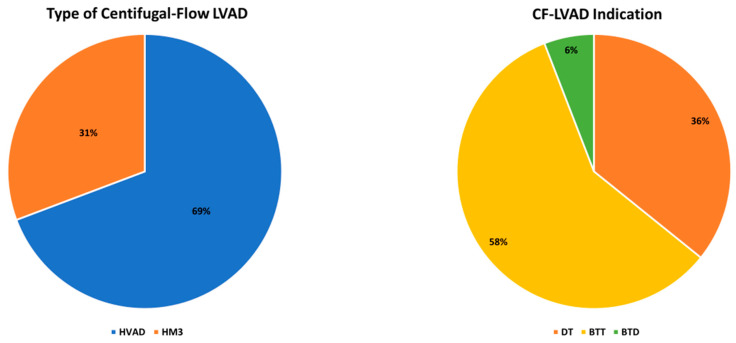
Centrifugal-flow left ventricular assist device type and indication.

Overall, 138 patients (67.7%) had at least one HF/LVAD-related readmission within three years post-LVAD implantation hospital discharge. Baseline demographic and clinical characteristics of patients with at least one HF/LVAD-related readmission vs. those without HF/LVAD-related readmission are presented in Table 2. Readmitted patients were comparable to non-readmitted patients in terms of demographics and past medical history, but they more commonly had undergone cardiac surgery in the past (23.9% versus 7.6%, *p* = 0.006). No differences were observed in the two patient subgroups in terms of disease duration and severity, as evidenced by HF symptoms duration, New York Heart Association classification and INTERMACS profile, inotrope dependence, and support with an intra-aortic balloon pump. However, patients with at least one HF/LVAD-related hospital readmission were less commonly supported with a microaxial percutaneous LVAD (Impella^®^; Abiomed, Danvers, MA, USA) or veno-arterial extracorporeal membrane oxygenation (VA-ECMO) prior to LVAD implantation compared to patients without (12.3% versus 25.8%, *p* = 0.026). Patients with an HF/LVAD-related readmission were more commonly supported with the HeartWare™ HVAD (Medtronic, Minneapolis, MN, USA) (75.4% versus 57.6%, *p* = 0.014) as opposed to the HeartMate 3™ LVAD (Abbott Laboratories, Chicago, IL, USA), while patients with at least one HF- or LVAD-related readmission had a longer duration on LVAD support (914.9 ± 725.3 versus 537.0 ± 570.4 days, *p* < 0.001), compared to non-readmitted patients. Of the overall cohort, 142/204 (69.6%) were supported with HVAD, and 119/204 (58.3%) patients received a centrifugal-flow LVAD as BTT (Figure 2).

Patients in both groups were similarly treated in terms of guideline-directed HF medical therapy and exhibited elevated cardiac filling pressures, severely impaired cardiac function, and abnormal cardiac structure prior to LVAD support. There were no differences in baseline hemodynamic, echocardiographic, and laboratory values between the two groups, apart from higher LVEF in patients readmitted for HF- or LVAD-related conditions (18.7 ± 7.6% versus 14.8 ± 6.7%, *p* < 0.001) and lower aspartate aminotransferase (AST) values (33.0 ± 24.5 versus 45.8 ± 56.1, *p* = 0.024). Intraoperative data, as well as hemodynamic and guideline-directed HF medical therapy data while on LVAD support, are presented in Table 3, with no significant differences being observed between the two patient groups.

**Figure 3:**
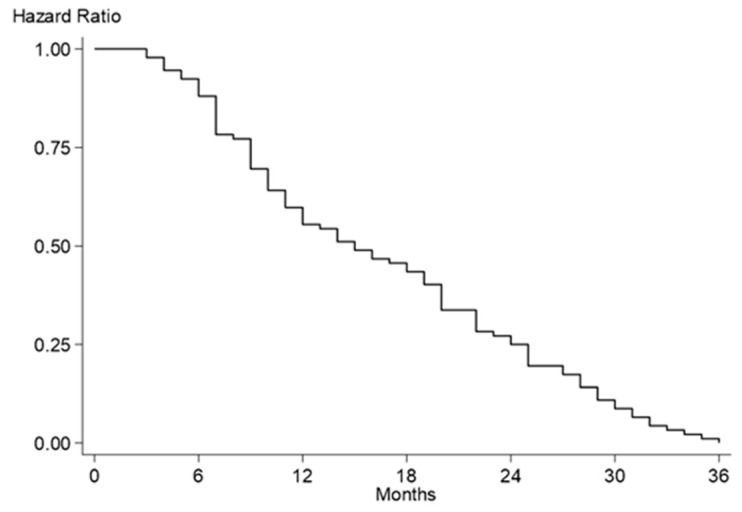
Hazard curve of heart failure/left ventricular assist device-related readmissions.

Two sets of multivariable Cox regression model estimates for HF/LVAD-related readmissions, with the first employing only baseline pre-LVAD implantation variables and the second also including intraoperative and post-LVAD implantation variables, are presented in Table 4. Estimates of the multivariable model employing only pre-LVAD implantation variables indicated that having at least one LVAD- or HF-related readmission was associated with pre-LVAD substance use, previous cardiac surgery, longer duration of HF symptoms, inotrope dependence, percutaneous LVAD/VA-ECMO support, use of a HeartWare™ LVAD, and higher LVEF (Table 4). The Harrell’s concordance c-statistic was 0.629. The multivariable model including intraoperative and post-LVAD implantation variables yielded the same eight variables as the first model plus LVAD implant hospitalization length of stay as an additional predictor of LVAD- or HF-related readmission (hazard ratio = 1.12, 95% confidence intervals = 1.09–1.14) and a similar Harrell’s concordance c-statistic of 0.640 (Table 4). Over the three years after LVAD implantation, the hazard of HF/LVAD-related readmission decreased steadily (Figure 3).

## 4. Discussion

Our findings suggest that readmissions due to HF- or LVAD-related reasons are common after LVAD implantation, with 67.7% of patients being rehospitalized at least once within three years post-LVAD implantation hospitalization discharge. HF- or LVAD-related readmissions accounted for 74.4% of all readmissions and the most common reasons included major bleeding, major infection, HF exacerbation, and neurological dysfunction. The hazard of HF- or LVAD-related readmissions decreased over the three-year period post-LVAD implantation hospital discharge.

It has been reported that 25–30% [4,5,6], 46% [3], and 71% [1] of patients are rehospitalized for any reason at 1, 3, and 12 months following LVAD implantation, respectively, by investigating patient data derived from large national registries. Furthermore, the national MCS registry reports that a large proportion of LVAD readmissions remain uncategorized (18.7%), which our study helps to further elucidate [1]. In our study, the rigorous, prospective follow-up of LVAD recipients allowed us to obtain a detailed picture of readmissions while on LVAD support and granular assessment of underlying readmission etiologies. Also, we focused our analysis on readmissions associated with the presence of LVAD or the underlying HF to avoid considering readmissions not necessarily related to the underlying disease, such as gastrointestinal, respiratory, and endocrine disorders, trauma, and planned procedures. Moreover, previous studies have focused on the early period (30 to 180 days) following device implantation [3,4,5,6]. We sought to investigate the reasons for readmissions during a longer follow-up period, especially given that most patients nowadays are implanted with a durable LVAD as DT [7]. Last, we focused our analysis on centrifugal-flow devices (HeartWare™ HVAD and HeartMate 3™ LVAD), which account for the vast majority of devices currently in use.

We found that the following pre-LVAD clinical factors were associated with HF or LVAD-related readmissions: substance use, previous cardiac surgery, duration of HF symptoms, pre-LVAD inotrope dependence, circulatory support with a percutaneous LVAD or VA-ECMO, support with the HeartWare™ HVAD, higher pre-LVAD LVEF, and lower AST values. After investigating intra- and post-operative variables alongside pre-operative variables, we found that a longer LVAD implantation hospitalization duration was further associated with the occurrence of HF/LVAD-related readmissions.

Some clinical factors are related to past medical and social history (history of substance use, previous cardiac surgery, and duration of HF symptoms), and although they are not actionable, they might inform risk assessment for HF/LVAD-related readmissions. Others reflect pre-LVAD disease acuity (pre-LVAD inotrope dependence and percutaneous LVAD/VA-ECMO support), while others relate to laboratory and imaging assessment (pre-LVAD LVEF and AST values). Previous cardiac surgery poses a technical challenge for LVAD implantation and has been suggested to increase the likelihood of readmissions within the first year post-LVAD implantation [10]. A longer duration of HF symptoms might suggest more advanced disease, but at the same time it can be linked to the potential for myocardial recovery. HF duration has been identified as a predictor of myocardial recovery [11,12,13,14], and recent data have indicated superior outcomes in patients with improved native left ventricular function on LVAD support, including HF rehospitalizations [15,16]. Pre-LVAD inotrope dependence and MCS with a percutaneous LVAD or VA-ECMO suggest more advanced disease, which might be accompanied by neurohormonal and end-organ derangements, affecting the probability of future readmissions. This might present an opportunity for improving post-LVAD outcomes and decreasing the number readmissions by means of durable LVAD implantation at an earlier disease stage, in agreement with previous data suggesting that post-LVAD outcomes in ambulatory advanced HF patients are superior compared to patients in cardiogenic shock or those who are inotrope-dependent [17]. Support with the HeartWare™ HVAD as opposed to the HeartMate 3™ LVAD was shown to increase the likelihood of future HF/LVAD-related readmissions, which might be consequential of a reportedly less favorable adverse event profile [18,19]. Lastly, a longer LVAD implantation hospitalization might be suggestive of medical or surgical complications increasing the chance of future readmissions.

The multivariable model identified pre-LVAD variables associated with post-LVAD hospital readmission. This model could potentially be utilized to identify a cohort of patients at higher risk for readmission and direct additional interventions, both preoperatively and post-LVAD. Transitional care interventions [20], inpatient rehabilitation [21], monitored performance improvement [22], and remote hemodynamic monitoring [23,24] are potential targets in patients at increased readmission risk that have been previously studied.

The limitations of the current study include the relatively small sample size and the inclusion of patients treated across collaborating sites (i.e., University of Utah Hospital and George E. Wahlen Department of Veterans Affairs Medical Center, Salt Lake City, Utah). Although the collaborative environment and research infrastructure allow for the rigorous, prospective follow-up of patients, they pose limitations regarding the generalizability of our findings. Similarly, although the lasso approach used for variable selection can prevent overfitting, we were not able to split the data into training and validation datasets to evaluate model performance due to the small sample size. Moreover, although we identified important clinical characteristics associated with readmission, additional clinical characteristics not captured as covariates in our models may have influenced the risk of readmission. Our study is limited to analyzing the risk of the first readmission post-LVAD implantation hospitalization discharge. Lastly, a significant limitation of this study is that the majority of the patients included were implanted with HVAD, which has an increased adverse events profile compared to the HM3 device and is no longer commercially available. This limits the generalizability of our findings to the current era of LVAD patients and warrants further investigation.

## 5. Conclusions

The twelfth Society of Thoracic Surgeons INTERMACS report highlighted the importance of the occurrence of readmissions while on LVAD support as an opportunity to further improve LVAD recipient outcomes. Our study findings confirm that rehospitalizations on contemporary centrifugal-flow LVAD support are a frequent and vexing clinical problem. HF- or LVAD-related readmissions accounted for most readmissions and were related to bleeding, infections, HF exacerbation, and neurological dysfunction. Multiple factors were found to be associated with readmissions including substance use, previous cardiac surgery, duration of HF symptoms, pre-LVAD inotrope dependence, pre-LVAD percutaneous LVAD/VA-ECMO support, LVAD type, pre-LVAD LVEF and AST values, as well as LVAD implant length of stay. Using machine learning-based techniques, we created models identifying patients with a high likelihood of rehospitalizations while on LVAD support employing pre-, intra-, and post-operative clinical variables. These models could serve as a guide to implement appropriate clinical strategies and focused care plans to mitigate this risk and further improve outcomes, as well as decreasing the burden on healthcare providers and systems.

## Figures and Tables

**Table 1:** Categorization of readmissions into heart failure/left ventricular assist device-related readmissions or not.

HF/LVAD-Related Readmissions	Non-HF/LVAD-Related Readmissions
Major bleeding	Gastrointestinal disorder
Major infection	Respiratory distress/failure
HF exacerbation	Trauma
Cardiac arrhythmia	Endocrine disorder
Device malfunction	Chest pain
Neurological dysfunction	Planned procedure
Anticoagulant adjustment	Other
Electrolyte abnormalities	
Acute or acute on chronic renal failure	
Pre-syncope/syncope	
Hypovolemia/hypotension	

HF: heart failure; LVAD: left ventricular assist device.

**Table 2:** Demographics and baseline clinical characteristics.

Variable	Overall (N = 204)	Non-HF/LVAD-Related Readmission (n = 66)	HF/LVAD-Related Readmission (n = 138)	*p*-Value
*Demographics*				
Age, years	55.7 (13.1)	54.6 (13.5)	56.3 (12.9)	0.399
Male sex, n (%)	171 (83.8)	56 (84.9)	115 (83.3)	0.842
Ethnicity and Race				0.129
Non-Hispanic White, n (%)	148 (72.5)	45 (68.2)	103 (74.6)	
Non-Hispanic Black or African American, n (%)	25 (12.3)	7 (10.6)	18 (13.0)	
Hispanic, n (%)	17 (8.3)	10 (15.1)	7 (5.1)	
Other, n (%)	14 (6.9)	4 (6.1)	10 (7.3)	
Body mass index, kg/m^2^	27.9 (5.9)	27.4 (5.6)	28.1 (6.0)	0.411
Body surface area, m^2^	2.0 (0.3)	2.0 (0.3)	2.0 (0.3)	0.729
*Medical and Social History*				
Smoking, n (%)	122 (59.8)	42 (63.6)	80 (58.0)	0.451
Ethanol use, n (%)	127 (62.3)	42 (63.6)	85 (61.6)	0.878
Substance use, n (%)	51 (25.0)	20 (30.3)	31 (22.5)	0.232
Diabetes mellitus, n (%)	81 (39.7)	25 (37.9)	56 (40.6)	0.761
Hypertension, n (%)	115 (56.4)	35 (53.0)	80 (58.0)	0.548
Atrial fibrillation, n (%)	90 (44.1)	29 (43.9)	61 (44.2)	0.972
Previous cardiac surgery, n (%)	38 (18.6)	5 (7.6)	33 (23.9)	0.006
Ischemic cardiomyopathy, n (%)	89 (43.6)	28 (42.4)	61 (44.2)	
NYHA class pre-LVAD				0.744
3, n (%)	61 (29.9)	21 (31.8)	40 (29.0)	
4, n (%)	143 (70.1)	45 (68.2)	98 (71.0)	
Duration of HF symptoms, months	86.0 (84.0)	80.1 (75.2)	88.8 (88.0)	0.247
Intermacs profile				0.177
1 or 2, n (%)	58 (28.4)	23 (34.8)	35 (25.4)	
3, n (%)	76 (37.3)	19 (28.8)	57 (41.3)	
4 or more, n (%)	70 (34.3)	24 (36.4)	46 (33.3)	
LVAD Type				0.014
HeartMate 3, n (%)	63 (30.4)	28 (42.4)	34 (24.6)	
HeartWare, n (%)	142 (69.6)	38 (57.6)	104 (75.4)	
LVAD Indication				0.664
Bridge to transplant, n (%)	119 (58.3)	39 (59.1)	80 (58.0)	
Destination therapy, n (%)	73 (35.8)	22 (33.3)	51 (36.9)	
Bridge to decision or recovery, n (%)	12 (5.9)	5 (7.6)	7 (5.1)	
Distance from implanting center, miles	568.7 (559.2)	527.4 (537.8)	588 (569.9)	0.467
LVAD implant length of stay, days	25.1 (18.2)	29.9 (22.1)	22.9 (15.5)	0.009
*Pre-LVAD Supportive Therapies*				
Inotrope dependence, n (%)	144 (70.6)	44 (66.7)	100 (72.5)	0.395
IABP, n (%)	25 (12.3)	8 (12.1)	17 (12.3)	0.968
pVAD/VA-ECMO, n (%)	34 (16.7)	17 (25.8)	17 (12.3)	0.026
*Pre-LVAD HF Medications*				
Beta-blocker, n (%)	127 (62.3)	43 (65.2)	84 (60.9)	0.644
ARNI/ACE-I/ARB, n (%)	123 (60.3)	43 (65.2)	80 (58.0)	0.361
Aldosterone blocker, n (%)	131 (64.2)	45 (68.2)	86 (62.3)	0.439
Diuretics, n (%)	188 (92.2)	59 (89.4)	129 (93.5)	0.404
*Pre-LVAD Hemodynamics*				
Mean right atrial pressure, mmHg	11.1 (6.3)	11.2 (5.9)	11.0 (6.5)	0.817
Pulmonary capillary wedge pressure, mmHg	23.9 (8.2)	24.3 (8.2)	23.7 (8.2)	0.595
Cardiac index by Fick, L/min/m^2^	1.9 (0.7)	1.8 (0.5)	2.0 (0.8)	0.174
*Pre-LVAD Laboratory Values*				
Hemoglobin, g/dL	12.1 (2.2)	12.2 (2.3)	12.0 (2.2)	0.477
Sodium, mEq/L	133.2 (5.6)	132.6 (6.2)	133.5 (5.3)	0.287
Potassium, mEq/L	4.1 (0.5)	4.0 (0.6)	4.1 (0.5)	0.513
Creatinine, mg/dL	1.4 (0.6)	1.4 (0.7)	1.4 (0.5)	0.952
Blood urea nitrogen, mg/dL	31.6 (16.3)	31.7 (16.8)	31.5 (16.1)	0.965
Aspartate transaminase, mg/dL	37.2 (38.0)	45.8 (56.1)	33.0 (24.5)	0.024
Alanine transaminase, mg/dL	48.6 (75.7)	60.4 (107.8)	43.0 (56.3)	0.126
Albumin, g/dL	3.7 (0.5)	3.7 (0.6)	3.7 (0.5)	0.601
Hemoglobin A1c, g/dL	6.3 (1.1)	6.2 (0.8)	6.3 (1.2)	0.569
*Pre-LVAD Echocardiographic Data*				
Left ventricular ejection fraction, %	17.5 (7.5)	14.8 (6.7)	18.7 (7.6)	<0.001
Left ventricular end-diastolic diameter, cm	6.6 (1.0)	6.6 (1.1)	6.6 (1.0)	0.973

ACE-I: angiotensin converting enzyme inhibitor, ARB: angiotensin II receptor blocker, ARNI: angiotensin receptor-neprilysin inhibitor, HF: heart failure, IABP: intra-aortic balloon pump, Intermacs: Interagency Registry for Mechanically Assisted Circulatory Support, LVAD: left ventricular assist device, NYHA: New York Heart Association, pVAD: percutaneous ventricular assist device, VA-ECMO: veno-arterial extracorporeal membrane oxygenation.

**Table 3:** Intra- and post-operative clinical characteristics.

Variable	Overall (N = 204)	Non-HF/LVAD-Related Readmission (n = 66)	HF/LVAD-Related Readmission (n = 138)	*p*-Value
*Intraoperative Data*				
Cardiac bypass duration, mins	88.2 (39.6)	92.8 (42.7)	86.0 (38.0)	0.253
Blood product units, n	4.1 (4.9)	4.4 (5.0)	3.9 (4.8)	0.515
* Post-LVAD HF Medications*				
Beta-blocker, n (%)	126 (61.8)	39 (59.1)	87 (63.0)	0.645
ARNI/ACE-I/ARB, n (%)	118 (57.8)	37 (56.1)	81 (58.7)	0.763
Aldosterone blocker, n (%)	119 (58.3)	40 (60.6)	79 (57.3)	0.762
Diuretics, n (%)	188 (92.2)	59 (89.4)	129 (93.5)	0.404
* Post-LVAD Hemodynamics*				
Mean right atrial pressure, mmHg	9.4 (5.2)	8.9 (5.2)	9.6 (5.2)	0.513
Pulmonary capillary wedge pressure, mmHg	13.1 (6.9)	24.3 (8.2)	23.7 (8.2)	0.366
Cardiac index by Fick, L/min/m^2^	2.4 (0.5)	2.4 (0.7)	2.4 (0.5)	0.855

ACE-I: angiotensin converting enzyme inhibitor, ARB: angiotensin II receptor blocker, ARNI: angiotensin receptor-neprilysin inhibitor, HF: heart failure, LVAD: left ventricular assist device.

**Table 4:** Multivariable model estimates for heart failure/left ventricular assist device-related readmissions.

Pre-Operative Multivariable Model				Pre-, Intra-, and Post-Operative Multivariable Model			
Variable	HR	95% CI	*p*-Value	Variable	HR	95% CI	*p*-Value
Substance Use	1.19	1.05–1.34	0.005	Substance Use	1.23	1.15–1.31	<0.001
Previous Cardiac Surgery	1.26	1.08–1.47	0.003	Previous Cardiac Surgery	1.26	1.13–1.41	<0.001
Duration of HF symptoms (quartiles)	1.10	1.07–1.14	<0.001	Duration of HF symptoms (quartiles)	1.12	1.09–1.15	<0.001
Pre-operative inotrope dependence	1.16	1.04–1.30	0.008	Pre-operative inotrope dependence	1.15	0.97–1.36	0.106
Pre-operative percutaneous LVAD/VA-ECMO	1.10	0.95–1.27	0.227	Pre-operative percutaneous LVAD/VA-ECMO	1.07	0.93–1.23	0.369
HeartWare™ HVAD	1.35	1.08–1.70	0.009	HeartWare™ HVAD	1.42	1.07–1.88	0.015
Pre-operative LVEF (quartiles)	1.12	1.11–1.13	<0.001	Pre-operative LVEF (quartiles)	1.13	1.10–1.15	<0.001
Pre-operative aspartate aminotransferase (quartiles)	0.98	0.97–1.00	0.05	Pre-operative aspartate aminotransferase (quartiles)	0.98	0.93–1.02	0.298
-	-	-	-	LVAD implant length of stay (days)	1.12	1.09–1.14	<0.001
Harrell’s concordance c-statistic = 0.629				Harrell’s concordance c-statistic = 0.640			

CI: confidence interval; HF: heart failure; HR: hazard ratio; LVAD: left ventricular assist device; LVEF: left ventricular ejection fraction; VA-ECMO: veno-arterial extracorporeal membrane oxygenation.

## Data Availability

All study data will be made available upon reasonable request to the corresponding author.

## Abbreviations

AST	Aspartate aminotransferase
BTT	Bridge to transplantation
CF	Centrifugal flow
DT	Destination therapy
HF	Heart failure
INTERMACS	Interagency Registry for Mechanically Assisted Circulatory Support
MCS	Mechanical circulatory support
LASSO	Least absolute shrinkage and selection operator
LVAD	Left ventricular assist device
LVEF	Left ventricular ejection fraction
RHC	Right heart catheterization
VA-ECMO	Veno-arterial extracorporeal membrane oxygenation
